# Workplace violence against homecare workers and its relationship with workers health outcomes: a cross-sectional study

**DOI:** 10.1186/s12889-014-1340-7

**Published:** 2015-01-17

**Authors:** Ginger C Hanson, Nancy A Perrin, Helen Moss, Naima Laharnar, Nancy Glass

**Affiliations:** Research Data and Analysis Center, Center for Health Research, Portland, Oregon USA; Labor Education and Research Center, University of Oregon, Portland, Oregon USA; Oregon Institute of Occupational Health Sciences, Oregon Health & Science University, Portland, Oregon USA; School of Nursing, Johns Hopkins University, Baltimore, Maryland USA

**Keywords:** Homecare, Workplace aggression, Workplace violence, Sexual harassment, Burnout

## Abstract

**Background:**

Consumer-driven homecare models support aging and disabled individuals to live independently through the services of homecare workers. Although these models have benefits, including autonomy and control over services, little evidence exists about challenges homecare workers may face when providing services, including workplace violence and the negative outcomes associated with workplace violence. This study investigates the prevalence of workplace violence among homecare workers and examines the relationship between these experiences and homecare worker stress, burnout, depression, and sleep.

**Methods:**

We recruited female homecare workers in Oregon, the first US state to implement a consumer driven homecare model, to complete an on-line or telephone survey with peer interviewers. The survey asked about demographics and included measures to assess workplace violence, fear, stress, burnout, depression and sleep problems.

**Results:**

Homecare workers (n = 1,214) reported past-year incidents of verbal aggression (50.3% of respondents), workplace aggression (26.9%), workplace violence (23.6%), sexual harassment (25.7%), and sexual aggression (12.8%). Exposure was associated with greater stress (p < .001), depression (p < .001), sleep problems (p < .001), and burnout (p < .001). Confidence in addressing workplace aggression buffered homecare workers against negative work and health outcomes.

**Conclusions:**

To ensure homecare worker safety and positive health outcomes in the provision of services, it is critical to develop and implement preventive safety training programs with policies and procedures that support homecare workers who experience harassment and violence.

**Electronic supplementary material:**

The online version of this article (doi:10.1186/s12889-014-1340-7) contains supplementary material, which is available to authorized users.

## Background

Our global population is aging; this is true for developed and developing nations alike [1]. Reasons for this trend include both declining fertility rates and increases in life expectancy. The current life expectancy at birth is now over 80 in 33 countries. Given the significance of this trend, there is a need for health care policies that will improve the quality of life for aging and disabled population, their family and those caring for them. The elderly and disabled have repeatedly expressed their desire to have control over care and remain active in their communities, therefore, in an effort to meet these appeals, health care funding policies in most western countries for long-term care for elders and disabled persons are shifting away from institutions, such as nursing homes and long-term care settings to the client’s home [2].

One approach innovative to homecare is the consumer-driven model in the U.S., or self-directed model as it is called in the UK [3]. The consumer-driven model funded through Federal/State entitlement programs, such as Medicaid/Medicare in the US, enables elderly or disabled individuals in need of supportive care to continue to live in their homes and communities while receiving support with activities of daily living (ADLs). Homecare workers in the consumer-driven model are employees of the consumer rather than an organization/institution. The homecare workers, often non-licensed workers, perform ADLs such as bathing and hygiene, dressing and grooming, eating, elimination, mobility and cognition/behavior support, as well as IADLs such as shopping, housekeeping, meal preparation, assistance with medication and oxygen and transportation for their employer for an assigned number of hours daily [4].

The consumer-driven homecare model has a variety of benefits for the consumer-employers and homecare workers. For consumer employers, the model supports the consumer’s autonomy and control over who is hired as their homecare worker and how the homecare worker implements support for the ADLs. Homecare workers report that they appreciate the informal work environment of the home, the ability to negotiate flexible work hours, and the meaningful relationships they can forge with their consumer-employers [5].

Although there are benefits, the consumer-employer and homecare worker relationship has the potential for safety challenges. Specifically, given the weak labor market position of homecare workers and their work in the consumer employer’s home, our previous research has demonstrated their vulnerability to sexual harassment and workplace violence [4]. These social and employment issues cannot be resolved in the same manner as employment health and safety issues within a hospital, clinic or nursing home setting where employees have access to employment assistance programs, human resources or security personnel. For homecare workers, the workplace is the consumer employer’s home and the perpetrator of sexual harassment and/or violence can be either the consumer employer or a relative or friend with the consumer employer. Further, limited training initiatives aimed to prevent or respond to sexual harassment and workplace violence are available to homecare workers, and consumer-driven program policies often do not specifically address sexual harassment and/or violence perpetrated by consumer employers or others in the home against homecare workers.

### Defining workplace violence

For our study, we used the definitions provided by Barling and colleagues [6], they defined four different types of workplace violence that homecare workers may experience: workplace aggression, workplace violence, sexual harassment, and sexual aggression. Workplace aggression refers to acts of non-physical aggression or threats of violence in the work setting (e.g. cornering someone, slamming a door, or threatening them with a weapon). Some studies also categorize verbal aggression (e.g., yelling, insulting, belittling) separately from workplace aggression [7-9] and we chose to follow this convention. Workplace violence refers to the occurrence of physical assault or physically threatening behavior (e.g., hitting with a fist or other object, kicking, biting, bumping with intentional force). Sexual harassment is defined as the occurrence of acts of a sexual nature that could be deemed offensive or intimidating, but were not physical acts (e.g., sexual comments, unwanted requests dates or sexual favors, leaving sexually explicit material in view). Sexual Aggression was defined as the occurrence of acts of a sexual nature involving physical contact (e.g., breaking personal boundaries, touching someone in a sexual way).

### Workplace violence in homecare

In the US, approximately 2 million workers are affected by workplace violence annually [10]. Workplace violence in healthcare and social services occupations has been recognized globally as a major occupational hazard [11-14]. Homicide is the number one cause of death in the workplace for nurses and employees in personal-care facilities [15]. Almost half of all non-fatal assaults in US workplaces occur in the healthcare or social service industries [14]. In the U.K., where a similar model of homecare is being implemented, assaults were among the top causes of workplace injuries resulting in 7 or more days of missed work in both the healthcare and residential care industries [16].

The threat of workplace violence is one of the top concerns of home healthcare workers, ranking higher than environmental hazards or transportation issues [17]. Several factors, including the lack of a large nationally representative sample and differences in methodology make it difficult to narrow in on the precise prevalence of workplace violence, but looking across several studies can offer some estimate. Survey results from several different studies have shown the percentage of homecare workers experiencing any form of workplace violence to be between 5-61% [7]. Verbal aggression is the most pervasive, reported by between 18-59% of homecare workers [6,7,17]: with the highest estimate coming from a study that ask about abuse over the homecare worker’s career [7] and the lower estimates coming from studies that ask about the occurrence in the last 6-months [6,17]. Workplace aggression, or threatening behavior were reported by 7-16% [6,7] of homecare workers, with the highest percentage coming from a study that asked about the occurrence over the homecare workers career [7], and lower percentage coming from a study that reported about the occurrence in the last 6 months [6]. Workplace violence or physical assaults were reported by between 2-11% of homecare workers [6,17,18], with the larger percentage coming from a broader definition of workplace violence that included being threatened with a knife [6], and the smaller percentages coming from studies that included general questions about physical assaults only [17,18].

Research has shown that workplace violence and sexual harassment and sexual aggression often co-occur [6]. A meta-analysis covering 86,578 participants from 55 separate probability samples across a variety of industries found that 58% of women report experiencing sexually harassing behaviors at work [19]. Nurses are believed to have a higher exposure to sexual harassment than many other occupations; studies have found that between 16-76% of nurses’ report experiencing sexual harassment over their careers [20-27]. Studies of homecare workers have found that approximately 30% of homecare workers reported being sexually harassed [6,28]. While reports of workplace violence and sexual harassment are high, scientists believe that the actual prevalence may be even higher given underreporting bias [29].

### Impact on work and health outcomes

Homecare workers’ experience of workplace violence and sexual harassment can impact their health both directly and indirectly. The most severe possible direct effect is homicide of the homecare worker [30], but more common direct effects are nonfatal injuries [31-33]. While the most severe forms of violence occur less frequently, even less-severe forms of workplace violence and sexual harassment are associated with a variety of negative outcomes for women’s physical and mental health [34]. The indirect personal impact of workplace violence on women’s health can be understood using the Lazarus and Folkman’s transactional stress and coping theory [35]. According to this perspective, experiences of workplace violence can overwhelm the homecare worker’s coping resources resulting in prolonged stress [36,37] and leading to poorer mental and physical health outcomes. Several studies have documented health effects of workplace violence on health outcomes, including depersonalization [38]; depression [18]; flashbacks, sleeplessness, poorer mental health [39]; traumatic stress disorder [40]; emotional exhaustion [38], and poorer physical health [38]. Health effects of workplace violence and harassment can last for years after the incident(s) [41].

Research has confirmed links between workplace violence and stressors such as fear of future violence [7,36,42-47], and has demonstrated that fear is a pathway by which workplace violence can affect health [6,36]. In addition, homecare workers do not need to experience workplace violence to report negative outcomes, as studies have shown that fear or perceived threat of workplace violence is associated with increased physical symptoms, anxiety, and poorer mental health [48]. Fear or perceived threat may be precipitated by witnessing or hearing about the negative experience of another homecare worker. Based on the transactional stress and coping theory, confidence in preventing and responding to workplace violence may be considered a resource that increases homecare workers capacity to cope with the stress and helps buffer the negative impacts on their health. A study conducted in one private homecare agency found that 93% of homecare workers were more confident after participating in violence-prevention training [49]. However, they did not go further to examine the impact of the increase in confidence on health outcomes.

### Purpose

**Figure 1:**
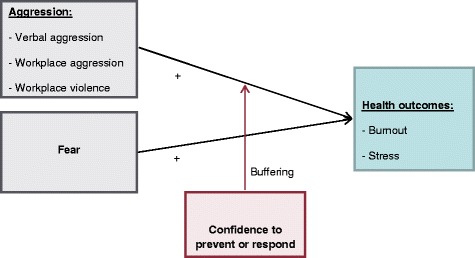
**Theoretical model of the relationships between forms of workplace aggression work and health outcomes.**

This study examines sexual harassment and workplace violence prevalence in a consumer-driven homecare model, where the potential outcomes for homecare workers who experience harassment and/or violence are not fully understood. We examined the prevalence of different types of workplace violence and sexual harassment as defined above, and the association of workplace violence, sexual harassment, and fear of violence or harassment on homecare worker’s work and health outcomes. Prevalence estimates are critical to supporting efforts of homecare workers and their advocates, such as labor unions, to develop training programs and policies to prevent sexual harassment and workplace violence. We also examined workers’ confidence in preventing and responding to sexual harassment and workplace violence as a moderator of the relationship between these experiences and negative work (e.g. burnout) and health (e.g. depression) outcomes, see Figure 1. This information is also important to developing homecare worker programs to reduce the negative outcomes often associated with experiencing harassment and violence.

## Methods

We used a cross-sectional design to explore the prevalence of workplace violence and sexual harassment experienced by homecare workers in a consumer-driven homecare model and to understand how these experiences related to homecare workers’ work and health outcomes. The study is in compliance with the Helsinki Declaration and received oversight and approval for the study from the IRBs at Johns Hopkins Medical Institutions (#20685) and Oregon Health and Science University (#4623). Our research was facilitated by a partnership with the Oregon Homecare Commission (OHCC) and with the Service Employees International Union (SEIU) Local 503, who also participated in our advisory board along with members of the study team and representatives for Oregon Department of Human Services DHS, homecare workers, and consumer-employers. The advisory board provided guidance on the development of the prevalence survey, statewide recruitment of homecare workers and safety protocol.

### Study participants

Eligibility criteria for the study included being female, able to speak English fluently, and currently employed as a homecare worker compensated through the Oregon Medicaid waiver program, or having worked as a homecare worker in the past 3 months. We focused on women because they make up about 86% of the homecare workforce in Oregon. Other studies have found differences in the reporting of workplace violence and sexual harassment experienced by men and women [34,50,51]. Given the under-representation of men in the population, our study did not have sufficient resources to recruit a large enough sample to adequately assess males’ experiences.

### Recruitment and data collection

We used a multi-pronged approach to recruitment. Participants were recruited from a roster of Oregon homecare workers supplied by SEIU Local 503. The initial list contained 11,836 homecare workers with some form of contact information. After removing duplicates and names that were highly likely to be male using an algorithm that compared the roster to a database of names from GenderChecker.com, we had a roster of 10,039 homecare workers. We then randomly selected 7,477 homecare workers for recruitment. After removing homecare workers that were unreachable (2,873) and those who did not meet the screening criteria (946), we were left with 3,658. Our final sample was 1,219, giving us a response rate of 33.32%. Homecare workers with an email address were sent a study invitation that explained the purpose, provided access to the web survey and the study phone number to ask questions of study members. They were sent up to three email reminders if they had not completed the survey after the initial invitation. Those homecare workers with no email address were sent a study brochure to their home address containing the same information as was in the email. If homecare workers did not respond to the emails or brochure, and they had a phone number listed they were transferred to a roster for phone outreach by peer interviewers. We made up to five outreach calls by phone. When we reached our recruitment goal (1,200) we stopped making outreach calls.

### Measurement

#### Workplace violence

We used an instrument developed by Barling et al. [6] to measure verbal aggression, workplace aggression, workplace violence, sexual harassment, and sexual aggression. Participants were asked to report on their experiences over the past year, including violence from their consumer-employer, or any other person in the consumer-employer’s home. We distinguished between verbal aggression and workplace aggression whereas Barling and colleagues’ classified both of these as workplace aggression. The participant was classified as experiencing verbal aggression if they answered yes to any of 3 items (example item: “in the past year, in your role as a homecare worker have you been yelled, shouted or sworn at?”) The workplace aggression was indicated if the participant had experienced any of 7 acts of non-physical aggression or threats of violence in the work setting (example item, “in the past year, in your role as a homecare worker have you had a door abruptly shut in your face?”) Workplace violence included 15 items asking about the occurrence of physical assault or physically threatening behavior in the work setting (example item, “in the past year, in your role as a homecare worker have you been kicked, bitten or hit with a fist?”) Sexual harassment occurred if the participant responded yes to any of 25 items that asked about the occurrence of acts or a sexual nature that could be deemed offensive or intimidating, but were not physical acts (example item, “in the past year, in your role as a homecare worker have you had sexually explicit material left in view?”). The participant was classified as experiencing Sexual Aggression if they indicated that any 11 acts of a sexual nature involving physical contact (example item, “in the past year, in your role as a homecare worker have you been touched in a sexual way?”) had occurred.

#### Fear

For the purpose of this study, fear is defined as the worry that one will experience some form of violence while working as a homecare workers. We measured fear by adapting the scale used by Barling et al. [6]. After each section of the questions (e.g., workplace aggression), participants were asked to indicate their agreement or disagreement with the statement, “I worry that I will experience workplace aggression while performing my duties as a homecare worker.” A similar question was asked after the sections on workplace violence, sexual harassment, and sexual aggression. These items were rated on a 5-point Likert-type scale ranging from 1 (strongly disagree) to 5 (strongly agree). We calculated a total score as the mean of these 4 items. The validity of the Barling et al. scale has been established in other studies with homecare workers [6,36]. The internal consistency, or Cronbach’s alpha, for these items was .90 in our sample.

#### Burnout

We used a subset of eight items from the work-related burnout and client-related burnout subscales of the Copenhagan Burnout Inventory (CBI) developed for the PUMA study [52]. The validity of this measure was established in a large sample of human service workers [53]. In our sample the two subscales used in the PUMA study were highly correlated. An exploratory-factor analysis indicated that there was a single factor, see Additional file 1. As a result, we collapsed them into a single work related burnout scale. Work related burnout can be defined as “a state of prolonged physical and psychological exhaustion, which is perceived as related to the person’s work” [54]. An example item is, “thinking about the last 4 weeks, is your work as a homecare worker emotionally exhausting?” The items were measured on a 5-point scale. We obtained a total score by taking the mean of the items and then rescaling so that the final score would range from 0–100. The Cronbach’s alpha for this scale was .9.

#### Stress, depression and sleep

We measured stress, depression, and sleep using the COPSOQ II [55]. Each subscale had four items. The introduction asked participants to think about how often in the past 4 weeks they had experienced each item due to working as a homecare worker. The developers of the COPSOQ II describe stress as a personal state characterized by both heightened arousal and displeasure. An example items is, “how often have you had problems relaxing?” The COPSOQ II measure of depression was designed to measure the level of depressive symptoms experienced by workers rather than to diagnose clinical depression. An example item is, “how often have you felt sad?” The sleep subscale is meant to be a measure of general sleeping troubles in a working population. An example item is, “how often have you found it hard to go to sleep?” All items were asked on a scale of 1 (not at all), 2 (a small part of the time), 3 (part of the time), 4 (a large part of the time) or 5 (all the time). We obtained a total score by taking the mean of the items and then rescaling so that the final score would range from 0–100. The validity of these sub-scales has been established by in previous research [56]. The Cronbach’s alphas for these scales were: α = .9_stess_, α = .8_depression_, and α = .9_sleep._

#### Confidence

We measured an individual’s confidence that she could prevent and respond to workplace violence and sexual harassment using a 19-item scale developed specifically for this study. We developed an initial list of items based on focus groups conducted by the study team with 83 homecare workers [4]. Then we sent these items to five subject-matter experts and asked them to rate the items from 0–2 on clarity, relevance, and usability. We retained items with a high mean on all three rating scales. The final rating scale for the items was 1 (not at all confident), 2 (a little confident), 3 (confident), or 4 (very confident). See Additional file 2 for final scale. Cronbach’s alpha for this scale was .9.

#### Covariates

Age was measured in number of years. Education was coded as 1 (8th grade or less), 2 (some high school), 3 (high school diploma or GED), 4 (some college), 5 (associate’s degree or vocational graduate), 6 (4 year college degree/bachelor’s degree), or 7 (post-Baccalaureate/Master’s degree/Ph.D). Tenure was coded as the number of years the participant has worked as a homecare worker. Hours worked was coded as the average number of hours worked weekly as a homecare worker. Additional jobs was coded as 0 (no additional jobs outside of homecare) or 1 (one or more jobs outside of homecare).

### Statistical analyses

We conducted three sets of analyses to answer the following questions: 1) what is the prevalence of different forms of workplace violence and sexual harassment among female homecare workers; 2) are experiences of workplace violence and fear related to negative work and health outcomes; and, 3) are these effects moderated by confidence in preventing and responding to workplace violence. We computed prevalence as the percent of respondents experiencing each item, and overall scores as the percent of respondents experiencing one or more items for each of the violence categories (i.e., verbal aggression, workplace aggression, workplace violence, sexual harassment, sexual aggression, and fear). Homecare workers providing services for their spouses were excluded from the sexual-harassment analyses. Scores for scales with more than 3 items were computed using mean replacement from the participant’s answered items if at least 75% of the questions were answered.

We used separate multiple regression analyses to regress each violence scale on each health outcome (stress, depression, and sleep) controlling for covariates. Poorer health outcomes are associated with increased age and lower socioeconomic [57,58], for this reason, age and education were included in all of the regression analyses to partial out any confounding effects. Burnout is known to be associated with work-related demographics [59]. Therefore, potential work related confounders including tenure, number of hours worked, and having additional jobs, were also included in the model when burnout was the outcome.

## Results

Table 1 shows demographic and work characteristics of the 1,214 homecare workers who completed the survey. Participants ranged in age from 19 to 80, with a mean age of 47.30 (SD = 13.8). The majority of homecare workers were White (85.4%), with 6.7% self-reported as Hispanic or Latina. Almost all of the participants (93.1%) had a high school diploma or GED, and 25.1% had a college or vocational degree. Participants reported having worked, on average, 7. 9 (SD = 7.3) years as a homecare worker. Twenty-one percent of participants lived with their consumer-employer. The average number of hours worked per week was 33.5 (SD = 27.6). Thirty-one participants worked for more than one consumer/employer, with the average working for between 1–2 consumer-employers (M = 1.5, SD = .8). The overwhelming majority of homecare workers provided services for someone other than their spouse at least part of the time (97.9%).

**Table 1:** **Description of the sample of homecare workers (N = 1214)**

	**N**	**%**
Race		
White	1027	85.4
Black or African American	44	3.7
Asian	20	1.7
American Indian or Alaskan Native	23	1.9
Native Hawaiian or Other Pacific Islander	6	0.5
Multi-racial	52	2.5
Other	30	4.3
Hispanic/Latina	81	6.7
Education		
8th grade or less	8	0.7
Some high school	76	6.3
High school diploma or GED	386	31.9
Some college	436	36.0
Associate’s degree or vocational graduate	192	15.9
Bachelor’s degree	85	7.0
Post-Baccalaureate/Master’s degree/Ph.D.	27	2.2
	**N**	**M (SD)**
Age	1136	47.3 (13.8)
Years worked as a HCW	1209	7. 9 (7.3)
Hours/week worked as a HCW	1213	33.5 (27.6)
Number of consumer-employers	1210	1.5 (.8)
	**N**	**%**
Works for 1 or more male consumer-employer(s)	472	39.1
Is an HCW for spouse only	26	2.1
Works at a job in addition to homecare worker	336	27.8
Experienced verbal aggression	611	51.5
Experienced workplace aggression	327	27.5
Experienced workplace violence	287	24.7
Experienced sexual harassment	312	27.6
Experienced sexual aggression	150	12.8
	**N**	**M (SD)**
Fear	1207	1.9 (1.0.96)
Burnout	1196	25.7 (23.0)
Stress	1206	26.1 (22.9)
Depression	1207	15.4 (17.9)
Sleep problems	1207	26.7 (26.2)
Confidence	1204	3.5 (.5)

### Prevalence

Table 2 summarizes prevalence of specific forms of verbal aggression, workplace aggression, workplace violence, sexual harassment, and sexual aggression in the last year. The percentage of homecare workers reporting one or more of these acts in the last year was as follows: verbal aggression (51.5%), workplace aggression (27.5%), workplace violence (24.7%), sexual harassment (27.6%), and sexual aggression (12.8%). Collapsing across all categories, 61.3% experienced at least one of these acts in the last year.

**Table 2:** **Prevalence of workplace violence towards homecare workers in the past year**

	**%**	**N**
**Verbal aggression**	**Yes**	**Yes**
Been yelled, shouted, or sworn at	41.6	496
Had someone be verbally aggressive to you	34.7	408
Had someone cry to make you feel guilty	29.2	351
**Workplace aggression**		
Been cornered or placed in a position that was difficult to get out of	18.6	223
Had a door abruptly shut in your face	11.3	135
Had someone try to hit you with something	9.3	112
Had someone harm themselves in front of you	6.5	78
Been threatened with a weapon other than a knife or a gun	2.2	26
Been threatened with a gun	0.8	9
**Workplace violence**		
Threat of violence (had someone threaten to throw something at you, hit you, had someone smash or kick something in your presence or display a loss of control)	20.8	248
Had someone try to hit you but failed, been kicked, bitten, hit with a fist, pushed, grabbed, shoved, or slapped	14.1	168
Been spat on or bumped with unnecessary force	9.1	108
Had your personal property damaged or destroyed (car, cell phone)	4.9	59
Had someone threaten to kill you	1.6	19
Had somebody handle a gun or a knife in a threatening way	1.6	19
Had someone fire a gun in your presence	0.4	5
Been choked	0.2	3
**Sexual harassment**		
Exposure to sexual explicit materials or comments	21.2	245
Sexual harassment (been target of rumors of sexual promiscuity, whistled or leered at, teased sexually, had sexual compliments)	16.6	191
Sexism (gender–based insults, sexist remarks)	13.9	161
Been asked personally intrusive question about your body or sex life	12.4	144
Received repeated requests for dates	3.5	41
Received sexual notes or other correspondence, been sexually propositioned (i.e., inited to engage in sexual intercourse)	3.1	36
Been offered money for sex	0.9	11
**Sexual aggression**		
Experienced someone breaking your personal boundaries, or been pinched, patted, hugged, or had an arm around you in a way that made you uncomfortable	11.3	134
Been fondled or touched in a sexual way	3.1	37
Had someone unnecessarily expose themselves in front of you	2.7	32
Been kissed in a way that made you feel uncomfortable	2.3	28
Had somebody physically restrain you	1.0	12
Been raped (e.g., forced to have sex against your will)	0.3	3

### Associations with work and health outcomes

We used multiple regression analyses to examine the relationships between each form of workplace violence and harassment and each health outcome separately, controlling for covariates. Table 3 presents the unstandardized regression weights for the effects of interest. A table of the correlations among all predictors and outcomes can be found in Additional file 3.

**Table 3:** **Multivariable regressions predicting health outcomes from different forms of workplace aggression and fear**

	**Unstandardized regression coefficients**			
**Model**	**Burnout**	**Stress**	**Depression**	**Sleep problems**
Verbal aggression	18.7	14.5	8.4	14.4
Workplace aggression	16.2	15.7	11.7	14.8
Workplace violence	18.5	15.4	11.8	16.0
Sexual harassment	14.6	14.7	9.0	11.7
Sexual violence	14.4	15.2	8.9	12.5
Fear	7.8	6.9	5.0	5.8

Notes. All regression coefficients were significant at the level of < .001. The covariates for burnout were age, education, tenure, hours, and additional jobs. The covariates for all other models were age and education. The scale for all health outcomes ranged from 0–100, where high scores indicate poorer health.

Experiencing any form of workplace violence or sexual harassment (i.e., verbal aggression, workplace aggression, workplace violence, sexual harassment, or sexual violence) was associated with greater stress, depression, and sleep problems among homecare workers controlling for age and education (see Table 3). For example, on a scale ranging from 0–100, participants who experienced verbal aggression scored, on average: 14.5 (*p* < .001) points higher on stress; 8.4 (*p* < .001) points higher on depression; and 14.4 (*p* < .001) points higher on sleep problems than participants who did not experience verbal aggression. Experiencing any form of workplace violence or sexual harassment was also associated with greater burnout controlling for age, education, tenure, hours, and additional jobs.

Our analyses show that fear of future workplace violence and sexual harassment was associated with worse health outcomes for homecare workers. Controlling for age and education, for every one-point increase in fear the average score on stress increased 6.9 (*p* < .001) points; depression increased 5.0 (*p* < .001) points; and sleep problems increased 5.8 (*p* < .001) points. For every one-point increase in fear, the average score on work burnout increased 7.8 (*p* < .001) points, controlling for age, education, tenure, hours, and additional jobs.

### Confidence to prevent and respond to violence and harassment as a moderator of negative work and health outcomes

**Figure 2:**
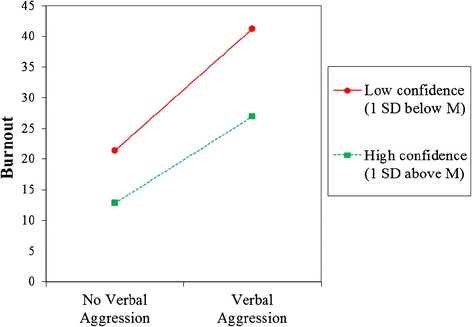
**Effects of verbal aggression and confidence on burnout.** Note. Lines are plotted at + and – 1 SD above and below the means for confidence. Regression formula: Burnout = 10.6 + 17.0(verbal aggression)-8.5(confidence)-5.6(verbal aggression*confidence)-.1(age) + 1.5(education) + .2(tenure) + .1(hours) + 2.1(additional jobs), R^2^ = .2.

**Figure 3:**
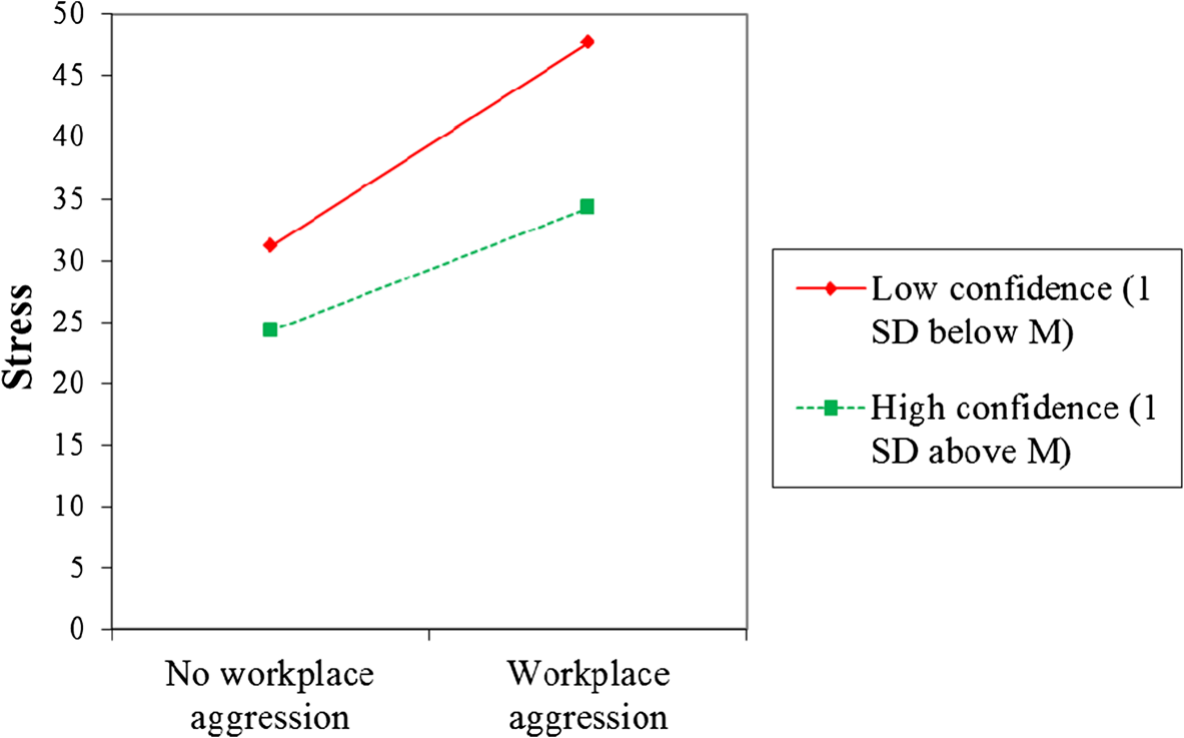
**Effects of workplace aggression and confidence on stress.** Note. Lines are plotted at + and – 1 SD above and below the means for confidence. Regression formula: stress = 30.0 + 13.3(workplace aggression)-6.8(confidence)-6.5(workplace aggression*confidence)-.2(age) + .7(education), R^2^ = .2.

To examine confidence as a moderator of the relationship between workplace violence/sexual harassment and health outcomes, the confidence variable (mean centered) and the interaction of the confidence with each workplace violence/sexual harassment variable were included in our multiple regression models. Controlling for covariates, confidence to prevent and respond to violence and harassment significantly buffered the effect of verbal aggression on burnout (B = −5.6, p = .023), and the effect of workplace aggression on stress (B = −6.5, p = .016). In other words, having higher confidence to prevent and respond to violence and harassment weakened the impact of verbal aggression on burnout, see Figure 2. In addition, having higher confidence to prevent and respond to violence and harassment weakened the impact of workplace aggression on stress, see Figure 3. None of the other interaction terms reached statistical significance.

## Discussion

### Key findings

Our findings indicate that homecare workers, a critical resource in a consumer-driven model of health care are experiencing substantial levels of workplace violence perpetrated by consumer-employers or other people in their home. Overall, 61.3% of female homecare workers in the consumer-driven model experienced at least one type of workplace violence in the past year. Our estimate of the prevalence of homecare workers experiencing verbal aggression (51.5%), workplace aggression (27.5%), or workplace violence (24.7%), sexual harassment (27.6%) and sexual aggression (12.8%) in this study is consistent with, or higher than, those of other studies [7,8,17]. The findings of this study add to the small but growing body of evidence that workplace violence is a serious occupational hazard for homecare workers.

Experiencing any form of workplace violence or fear of violence was associated with negative work and health outcomes. Specifically, experiencing verbal aggression, workplace aggression, workplace violence, sexual harassment, sexual aggression, or greater fear was associated with more work burnout, stress, depression and sleep problems. Our study provided mixed evidence that confidence in addressing these problems can buffer the impact of workplace violence and sexual harassment. Sp ecifically, confidence to prevent and respond to violence and harassment buffered the effect of verbal aggression on work burnout and the effect of workplace aggression on stress. However, in the relationship between other forms of workplace violence/sexual harassment and health outcomes, confidence did not act as a buffer.

Previous studies examine the role of personal resources such as confidence as a buffer of the negative effect of stress on health [60]. Social-learning theory suggests that fear results from a lack of self-efficacy about one’s ability to cope with potentially harmful events [61]. Research with homecare workers has indicated that fear of future harassment/violence is a pathway by which harassment and workplace violence affects health [6,36]. Confidence can play a role in reducing the autonomic response to fear before, during, or after a stress-inducing experience [61]. If one is confident that she can prevent, stop, or reduce the severity of the event, there is little reason to fear it. We did find some evidence that confidence buffered the effect of verbal aggression on burnout and workplace aggression on stress. However, we were not able to find evidence that confidence buffered the effect of workplace violence, sexual harassment, or sexual aggression. Our mixed results may be partly attributable to the low variability of our confidence scale, which was somewhat limited by a ceiling effect.

### Implications

The presence of a stable and healthy workforce will make it easier for consumer - employers to receive continuous high quality care [7]. The US Department of Labor projects that more than 1.3 million jobs will be added in this industry, a 70% increase from 2010 to 2020 [62]. The UK had approximately 1.56 million people employed in the adult social care workforce in 2012. By 2025 it is projected that the adult social care workforce could increase to as many as 2.86 million workers [63]. This growth is driven by the aging of baby boomers, increase in life expectancy, and a growing value placed on consumer-centered care [64], and the desire to lower healthcare costs for recovery and long-term care. Our study found that experiencing workplace violence and/or sexual harassment is associated with work burnout. Other research with health care providers have found similar result, studies indicate that nurses who experienced higher levels of burnout were more likely to express intentions to leave the profession [46,65]. Studies have also shown that workplace violence is directly related to increased turnover [17,47]. Furthermore, workplace violence or sexual harassment may interfere with interpersonal interactions between the consumer-employer and their homecare worker, directly reducing the quality of care. One study found that 68% of homecare workers would cut short a visit with a consumer-employer if they felt unsafe in the home [66]. Thus, addressing workplace violence can benefit both homecare workers by reducing burnout and consumer-employers by creating a work environment that is safe and allows high quality services to be provided by the homecare worker.

It is important to note that not only the most severe forms of workplace violence are related to poorer health; verbal aggression and non-physical aggression are also associated with poorer health outcomes for homecare workers. Other studies have found non-physical violence to be strongly related to negative health outcomes for employees [47], possibly because verbal aggression and non-physical violence are more pervasive. In addition, it is not just the experience of workplace violence or sexual harassment that impacts health, but also the fear of experiencing violence and harassment at work that impacts work and health outcomes. This findings confirms evidence from other research; employees who have never experienced workplace violence, but who fear or perceive a threat of experiencing workplace violence, may experience increased physical symptoms, anxiety, and poorer mental health [48].

### Policy recommendations

Policies and procedures for the consumer-driven homecare programs must balance the need for autonomy and independence of the consumer-employer against the workplace safety of homecare workers (and consumer-employers). Studies indicate that the consumer-driven model may expose homecare workers to harassment and violence given the lower levels of home monitoring and supervision, as compared to the agency-based home care models. Consumer-driven models require protocols and training for both consumer-employers and homecare workers to collaboratively assess for potential safety risks at the initial employment interview and throughout the service relationship as health and social conditions may change. Increasing the consumer employers and homecare workers knowledge, skills and resources to effectively prevent and respond to harassment and violence will likely increase the homecare workers confidence and reduce their fear of future harassment and violence, likely improving services to the employer consumer and preventing negative work and health outcomes for the homecare worker [15].

Policies for consumer-driven models should clearly state to consumer-employers and homecare workers that workplace violence and sexual harassment will not be tolerated [67]. Consequences for violating such policies should also be clearly delineated for consumer employers and homecare workers. Sanctions for use of threats, violence and harassment of homecare workers may increase consumer employers’ motivation to exercise restraint with regard to their role as employer and supervisor [68]. Existing procedures for reporting and investigating reports of workplace violence or other high-risk behaviors such as substance abuse, should be examined for gaps and strengthened where needed.

For example, under Oregon’s consumer-driven model, a homecare worker leaving the home of a person who requires 24/7 care is considered abandonment and can result in the loss of the homecare worker’s provider number (which can never be reinstated) and loss of employment. During the study period, the Oregon Homecare Commission added a provision to allow the homecare worker to leave if she or he felt at risk of serious injury. When establishing such provisions, it is important to also provide clear guidelines on appropriate procedures for leaving the home (e.g., notifying the consumer-employer’s family/emergency contact, and/or requesting a welfare check by the local police) and on documenting the situation/use of the procedures so that if the homecare workers actions are called into question, there is a record.

### Consumer-employers

An important strategy to support consumer employers in their role as employers and supervisors of homecare workers is to expand or create training program to emphasize definitions of workplace violence and sexual harassment [12]. Training for consumer-employers could also include: interviewing techniques; questions to ask related to safety; skills to establish work boundaries; and techniques to resolve conflicts that may arise in scheduling, work expectations, or performance. This training would, ideally, be mandatory for all consumer employers.

### Homecare workers

Due to the lack of organizational (supervisor and coworker) support, homecare workers are largely dependent on their own knowledge and skills to keep them safe. For this reason, training is extremely important. In other research, when asked to rank resources that would minimize homecare workers risks in their workplace, instituting safety programs ranked in the top three choices of both homecare workers and administrators [66]. Topics appropriate for the training should include: workplace policies and procedures, legal issues, identifying warning signs of violence, safety planning, assertive communication, conflict resolution, de-escalation of conflict, and self-care [29,69,70].

### Limitations

Our study design was cross-sectional, which was an adequate and efficient way to assess prevalence, but precludes our ability to determine a causal direction of the relationship between workplace violence, sexual harassment, and health outcomes. Our 33% response rate was not unexpected given some of the barriers to reaching this population, such as income and housing insecurity, that impact their access to consistent and reliable phone and internet services. Given this response rate, we cannot rule out the possibility that respondents may have been more likely to experience workplace violence than non-respondents. However, we were careful during recruitment to emphasize the importance of hearing from all workers, whether or not they had experience workplace violence. There is also the possibility that participants may under-report exposure, wanting to give socially desirable responses to sensitive questions. Healthcare workers tend to under-report violent incidents, in part because they see such incidents as “part of the job” [71,72]. Also, while we eliminated participants providing services for only their spouses from the sexual-harassment analyses, we did not ask whether a homecare worker was providing services to an intimate partner or ex-intimate partner. However, we do not suspect that asking about intimate or ex-intimate partners would have increased significantly the small number of homecare workers (2.14%) who provided services only to a spouse or partner. This assumption is supported by the percentage of homecare workers in our study reporting sexual harassment, which is similar to, if not lower than, other studies we reviewed [21-23,25,26]. Finally, the focus of this study was on homecare workers from Oregon’s consumer-driven model, thus the findings may not be generalizable to homecare workers working under different models, such as private or agency-based models.

## Conclusions

As our global population ages, the importance of retaining a health workforce of homecare workers is of increasing importance. Homecare models similar to Oregon’s consumer-driven model are exist in the UK and in several US states including California, Connecticut, Illinion, Maryland, Massachusetts, Minnesota, Missouri, and Washington. Given this, we feel that our study findings may be useful to policy makers in a wide variety of locations who are currently using or may be considering a similar model of homecare. Our research adds to the literature demonstrating that homecare workers are at high risk of exposure to incidents of workplace violence and sexual harassment, and that these experiences are related to increased stress, depression, burnout, and sleeping problems. In order to ensure homecare worker safety and positive health outcomes for both worker and consumer-employer, it is necessary to develop preventive safety policies and procedures and provide prevention training. More research is needed to understand how best to intervene to reduce homecare workers’ exposure to workplace violence and sexual harassment. Homecare worker trainings should be designed to increase confidence and capacity to plan for safety, establish and maintain appropriate work boundaries, and de-escalate violence and harassing situations.

## Additional files

Additional file 1:
**Exploratory factor analysis on burnout.**
Additional file 2:
**Confidence in preventing and responding to sexual harassment and workplace violent scale.**
Additional file 3:
**Correlations among covariates, predictors, moderators, and outcomes.**
