# Supplementary material for: Workplace violence against homecare workers and its relationship with workers health outcomes: a cross-sectional study
Source: BMC Public Health. 2015 Jan 17;15:11. doi: 10.1186/s12889-014-1340-7 (PMC4308913; doi:10.1186/s12889-014-1340-7)
Supplement: Additional file 1: — Exploratory factor analysis on burnout. [file 12889_2014_1340_MOESM1_ESM.doc]

**Additional file 1**

**Exploratory factor analysis on burnout**

This study used a subset of eight items from the work-related burnout and client-related burnout subscales of the Copenhagan Burnout Inventory (CBI) developed for the PUMA study [52]. In our sample the two subscales were highly correlated. For this reason we conducted an exploratory-factor analysis using iterative principle axis factoring. Only the first factor had an eigenvalue above 1 (5.20). This factor accounted for 60.31% of the variance in the individual items. Given these results the single factor solution was chosen. The extracted communalities and factor loadings are displayed in Table 1.1.

**Table 1.1 - Factor Loadings and Extracted Communalities for Burnout items**

|  | Extracted  Communalities | Factor  Loading |
| --- | --- | --- |
| Do you feel worn out at the end of your work day as a homecare worker? | .50 | .71 |
| Are you exhausted in the morning at the thought of another day at work as a homecare worker? | .72 | .85 |
| Do you feel that every working hour as a homecare worker is tiring for you? | .61 | .78 |
| Do you sometimes wonder how long you will be able to continue working with your consumer employer(s)? | .43 | .66 |
| Is your work as a homecare worker emotionally exhausting? | .69 | .83 |
| Does your work as a homecare worker frustrate you? | .65 | .81 |
| Do you feel burnt out because of your work as a homecare worker? | .77 | .88 |
| Do you feel that you give more than you get back when you work with your consumer employer(s)? | .46 | .68 |
