# Supplementary material for: Workplace violence against homecare workers and its relationship with workers health outcomes: a cross-sectional study
Source: BMC Public Health. 2015 Jan 17;15:11. doi: 10.1186/s12889-014-1340-7 (PMC4308913; doi:10.1186/s12889-014-1340-7)
Supplement: Additional file 2: — Confidence in preventing and responding to sexual harassment and workplace violent scale. [file 12889_2014_1340_MOESM2_ESM.doc]

**Additional file 2**

**Confidence in preventing and responding to sexual harassment and workplace violent scale**

Rating scale: 1=Not at all confident, 2=A little confident, 3=Confident, 4=Very confident

How confident are you that:

1. Could break a hold if a consumer employer or family friend of consumer employer grabbed you and wouldn't let go
2. Understand your legal rights if you should face violence or sexual harassment on the job
3. Could take action (e.g., leave, call for help) when you felt that you are in a situation that may not be safe
4. Would know who to call if your consumer employer or someone else in their household was hurting or threatening to hurt you
5. Know the steps to take if you need to leave your consumer employers home due to an abusive situation
6. Can maintain established work boundaries with your consumer employer
7. Could change the subject if your consumer employer was verbally abusive or made sexually suggestive comments
8. Could redirect the attention of a consumer employer when he she was speaking or acting in a way that made you uncomfortable
9. Could defuse a dispute over doing tasks that were not on the task list
10. Could redirect your consumer employer if you could see that they were becoming upset
11. Could tell a consumer employer that their sexual advances were not wanted or appropriate
12. Could tell a consumer employer to stop what they are doing when they are hurting you or touching you in an inappropriate way
13. Could talk with your consumer employer about his her violence and or sexual harassment if you needed to
14. That you could recognize factors (e.g., interruptions in routines, difficult transitions, mood disturbances, mental illness) that might lead to problem behaviors by your consumer employer
15. That you could determine when comments or behaviors are abusive
16. That you would know what to do if your consumer employer was exposing you to unwanted material or behavior of a sexual nature
17. That you would know when to call 911 versus the local office when you feel unsafe
18. That you could report abusive behavior by the consumer employer to the local office
19. That you know what to do if the case manager does not respond to your report of abuse by a consumer employer
