# Supplementary material for: Workplace violence against homecare workers and its relationship with workers health outcomes: a cross-sectional study
Source: BMC Public Health. 2015 Jan 17;15:11. doi: 10.1186/s12889-014-1340-7 (PMC4308913; doi:10.1186/s12889-014-1340-7)
Supplement: Additional file 3: — Correlations among covariates, predictors, moderators, and outcomes. [file 12889_2014_1340_MOESM3_ESM.doc]

**Additional file 3**

**Table 3.1 – Correlations among covariates, predictors, moderators, and outcomes**

|  | 1 | 2 | 3 | 4 | 5 | 6 | 7 | 8 | 9 | 10 | 11 | 12 | 13 | 14 | 15 |
| --- | --- | --- | --- | --- | --- | --- | --- | --- | --- | --- | --- | --- | --- | --- | --- |
| Age |  |  |  |  |  |  |  |  |  |  |  |  |  |  |  |
| Education | .06^*^ |  |  |  |  |  |  |  |  |  |  |  |  |  |  |
| Tenure | .32^**^ | -.08^**^ |  |  |  |  |  |  |  |  |  |  |  |  |  |
| Hours | -.06^*^ | -.05 | -.02 |  |  |  |  |  |  |  |  |  |  |  |  |
| Additional jobs | -.08^**^ | .17^**^ | -.02 | -.14^**^ |  |  |  |  |  |  |  |  |  |  |  |
| Verbal aggression | -.04 | .03 | .00 | .09^**^ | -.05 |  |  |  |  |  |  |  |  |  |  |
| Workplave aggression | -.02 | .02 | .03 | .09^**^ | -.01 | .52^**^ |  |  |  |  |  |  |  |  |  |
| Workplace violence | .02 | .03 | .04 | .11^**^ | -.03 | .50^**^ | .59^**^ |  |  |  |  |  |  |  |  |
| Sexual harrassment | .01 | .06 | .04 | .07^*^ | -.04 | .40^**^ | .38^**^ | .42^**^ |  |  |  |  |  |  |  |
| Sexual violence | -.02 | .05 | .00 | .03 | .02 | .28^**^ | .37^**^ | .37^**^ | .52^**^ |  |  |  |  |  |  |
| Fear | .00 | .08^**^ | .04 | .04 | -.02 | .36^**^ | .36^**^ | .35^**^ | .39^**^ | .35^**^ |  |  |  |  |  |
| Confidence | .04 | -.10^**^ | .08^**^ | .06^*^ | .04 | -.13^**^ | -.18^**^ | -.15^**^ | -.22^**^ | -.19^**^ | -.31^**^ |  |  |  |  |
| Burnout | -.06^*^ | .11^**^ | .01 | .10^**^ | .02 | .41^**^ | .31^**^ | .35^**^ | .28^**^ | .22^**^ | .32^**^ | -.30^**^ |  |  |  |
| Stress | -.14^**^ | .06^*^ | -.03 | .12^**^ | -.04 | .31^**^ | .29^**^ | .28^**^ | .27^**^ | .22^**^ | .29^**^ | -.26^**^ | .61^**^ |  |  |
| Depression | -.11^**^ | .08^**^ | -.05 | .11^**^ | -.04 | .23^**^ | .28^**^ | .27^**^ | .22^**^ | .17^**^ | .26^**^ | -.31^**^ | .57^**^ | .74^**^ |  |
| Sleep problems | -.07^*^ | -.01 | -.03 | .16^**^ | -.07^*^ | .26^**^ | .24^**^ | .25^**^ | .18^**^ | .15^**^ | .21^**^ | -.13^**^ | .45^**^ | .75^**^ | .63^**^ |

*. Correlation is significant at the 0.05 level (2-tailed).

**. Correlation is significant at the 0.01 level (2-tailed).
